# GADD45β, an anti-tumor gene, inhibits avian leukosis virus subgroup J replication in chickens

**DOI:** 10.18632/oncotarget.12027

**Published:** 2016-09-15

**Authors:** Xinheng Zhang, Zhuanqiang Yan, Xinjian Li, Wencheng Lin, Zhenkai Dai, Yiming Yan, Piaopiao Lu, Weiguo Chen, Huanmin Zhang, Feng Chen, Jingyun Ma, Qingmei Xie

**Affiliations:** ^1^ College of Animal Science, South China Agricultural University & Guangdong Provincial Key Laboratory of Agro-Animal Genomics and Molecular Breeding & Key Laboratory of Chicken Genetics, Breeding and Reproduction, Ministry of Agriculture, Guangzhou, 510642 P. R. China; ^2^ Key Laboratory of Animal Health Aquaculture and Environmental Control, Guangdong, Guangzhou 510642, P. R. China; ^3^ South China Collaborative Innovation Center for Poultry Disease Control and Product Safety, Guangzhou 510642, P. R. China; ^4^ USDA, Agriculture Research Service, Avian Disease and Oncology Laboratory, East Lansing, MI 48823, U.S.A

**Keywords:** ALV-J, RNA-Seq, differentially expressed genes, GADD45β, viral replication

## Abstract

Avian leukosis virus subgroup J (ALV-J) is a retroviruses that induces neoplasia, hepatomegaly, immunosuppression and poor performance in chickens. The tumorigenic and pathogenic mechanisms of ALV-J remain a hot topic. To explore anti-tumor genes that promote resistance to ALV-J infection in chickens, we bred ALV-J resistant and susceptible chickens (F3 generation). RNA-sequencing (RNA-Seq) of liver tissue from the ALV-J resistant and susceptible chickens identified 216 differentially expressed genes; 88 of those genes were up-regulated in the ALV-J resistant chickens (compared to the susceptible ones). We screened for significantly up-regulated genes (*P* < 0.01) of interest in the ALV-J resistant chickens, based on their involvement in biological signaling pathways. Functional analyses showed that overexpression of GADD45β inhibited ALV-J replication. GADD45β could enhance defense against ALV-J infection and may be used as a molecular marker to identify ALV-J infections.

## INTRODUCTION

Avian leukosis viruses (ALVs) are species of the *Alpharetrovirus* genus (family: Retroviridae) [1, 2]. Six subgroups of ALV are known in chickens, referred to as ALV-A, -B, -C, -D, -E, and -J. ALV-J is an oncogenic exogenous retrovirus of chickens; it was first reported in the United Kingdom in 1991, and has subsequently caused severe economic losses in the poultry industry worldwide [3, 4]. Hosts with clinical ALV-J infections develop of a variety of tumors, including myelocytomas, sarcomas, hemangiomas, nephromas and erythroblastosis and have the characteristic of delayed growth, high mortality, and immune tolerance [5, 6, 7, 8]. ALV-J can be transmitted vertically and horizontally, and horizontal transmission of ALV-J is more efficient than for other ALV subgroups [9]. The control and eradication of ALV-J from pedigree generations has become a priority in the primary breeder industry [10].

Analyzing host gene expression profiles following ALV-J infection is used to elucidate the molecular pathogenesis of ALV-J infection and tumor development [11, 12]. High-throughput mRNA sequencing (RNA-Seq) and real-time PCR are used to assess mRNA expression in what is a standardized approach, with high accuracy, sensitivity and reproducibility; thus, these methods have been widely used to infer host gene expression in response to virus infection [13, 14].

GADD45β is a member of the growth arrest and DNA damage 45 (GADD45) family of small (18 kDa) proteins. These proteins are associated with cell growth control, apoptotic cell death, and the cellular response to DNA damage [15]. GADD45β is necessary for full expression of the Th1 lineage-inducing proteins which include T-bet, and Eomes, and is important for anti-tumor immune responses [16]. When CD40, a TNF receptor superfamily member, ligates with GADD45β and known anti-apoptotic proteins, such as Bcl-XL and c-FLIP provide co-stimulatory signals to B cells [17]. Various human tumor studies have shown that GADD45β was aberrant and while GADD45β has clear features of tumor suppression. Because of its roles in DNA repair and cell growth, GADD45β might also offer survival advantages to certain malignant cells. GADD45β is also an anti-apoptotic protein in B cells, since it protects them from aberrant, activation-induced cell death [18]. GADD45β gene expression is suppressed by a variety of mechanisms in different tumors and tumor cell lines; the tumor suppressor function of GADD45β was demonstrated by an reduced colony formation, inhibition of growth and increased apoptosis, when it was overexpressed in the LBT2 mouse gonadotrope cell line [19].

To date, there have been no studies into the potential of eradicating ALV-J from its host via anti-disease genes. To look for anti-viral or anti-tumor genes and molecular markers, we chose chickens from eight lines and infected each with ALV-J, to identify the chicken line that was most resistant to ALV-J infection. Then we challenged chickens in that line with ALV-J for three generations. In each generation, we detected the ALV-J infection rate using four methods every 2 weeks, to identify whether the chickens had congenital resistance or susceptibility to ALV-J infection. Finally, we collected liver samples from the ALV-J infection resistant and susceptible chickens in the third generation. RNA-Seq was used to screen for differentially expressed genes and identify possible anti-viral genes. Our study provides the foundations for research into the mechanisms behind the pathogenicity and tumorigenicity of ALV-J, and a basis for breeding disease resistant chicken lines.

## RESULTS

### Resistance of chicken lines to ALV-J infection

Chickens in the A, E, and F lines were more resistant to ALV-J; the positive rates of infection with ALV-J in the A, E, and F lines were 16.49% (16/97), 36.84% (35/95), and 35.79% (34/95), respectively. The chickens in the B, C, D, G, and H lines were more easily infected with ALV-J; the positive infection rates were 80.41% (78/97), 100% (95/95), 97.98% (97/99), 59.79% (58/97), and 100% (91/91) respectively. Because of their high resistance to ALV-J infection, chickens from the A line were used for the rest of the study.

### mRNA expression profiles

**Figure 1:**
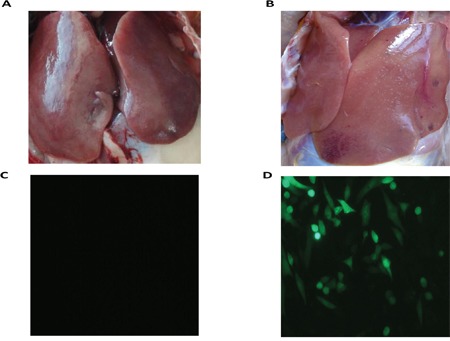
RNA-Seq sample **A.** Liver of resistant chicken; **B.** liver of susceptible chicken; **C.** determination of ALV-J infection of resistant chicken by IFA after inoculating liver homogenate into DF-1 cells; **D.** determination of ALV-J infection of susceptible chicken by IFA after inoculating liver homogenate into the DF-1 cells.

**Figure 2:**
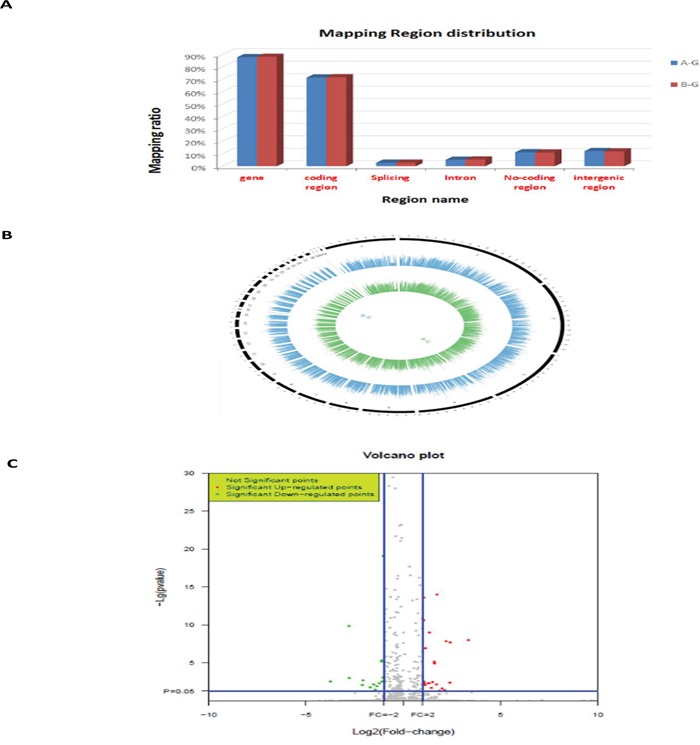
RNA-Seq data **A.** Mapping regions distribution within read alignments; **B.** genome coverage distribution of 1kh windows (outline is the reference genome, inside is the chromosome coverage across all samples); **C.** distribution of differentially expressed genes in ALV-J resistant (compared to susceptible) chickens. A-G: susceptible chickens; B-G: resistant chickens.

There were differences in the samples of the liver from the ALV-J-resistant and -susceptible chickens in the F3 generation of the A line, after challenge with ALV-J. The livers of ALV-J resistant chickens were normal, while livers of susceptible chickens showed swollen lesions, hemorrhagic spots and white nodules, livers of susceptible chickens were also found to be positive for infection by indirect immunofluorescent assay (IFA) (Figure 1). RNA-Seq was used to profile the mRNA expression levels in the livers of the ALV-J-resistant and -susceptible chickens after challenge with ALV-J. The RNA quality assessment (using Agilent Bioanalyzer 2100 electrophoresis) showed RNA Integrity Numbers of 9.3 and 9.4, for the ALV-J resistant and susceptible chickens, respectively. In total, 14,717,236 reads and 15,478,370 reads were obtained from the ALV-J-resistant and-susceptible chickens, respectively (Table 1). A BLAST search, using the chicken reference genome, produced 12,061,966 and 12,728,550 mapped reads for the ALV-J-resistant and -susceptible chickens, respectively, with mapped ratios of 82.2% and 82% (Figure 2).

**Table 1:** Genome sequences alignment of susceptible chickens and resistant chickens

Sample	Total reads	Mapped reads	Pair mapped reads	Single mapped reads	Unique mapped reads	Multi mapped reads	Mapped ratio
A-G	15,478,370	12,728,550	11,822,182	906,368	12,398,189	330,361	82.2%
B-G	14,717,236	12,061,966	11,213,872	848,094	11,739,071	322,895	82.0%

A-G: susceptible chickens; B-G: resistant chickens.

### Differentially expressed genes

**Figure 3:**
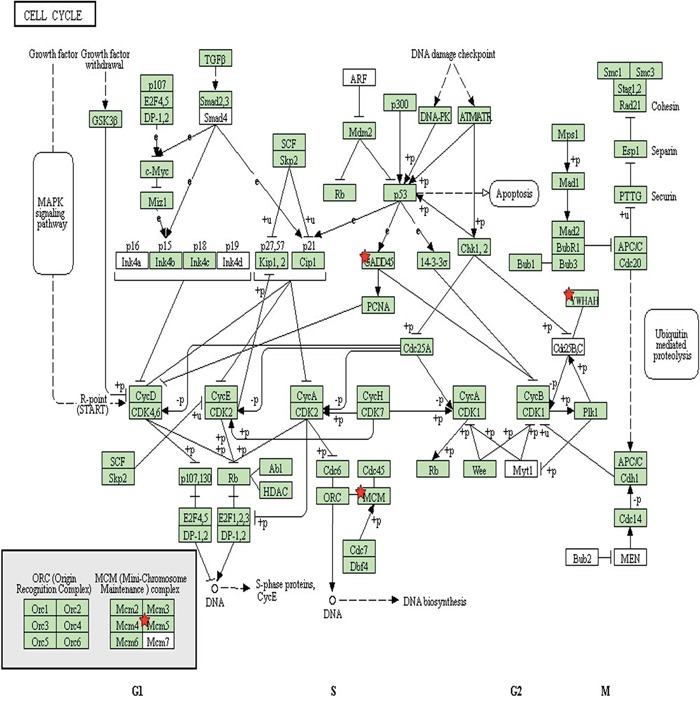
Signaling pathway analysis of proteins involved in the cell cycle. Red stars indicate three of the proteins of interest (GADD45β, MCM5 and YWHAH) identified in this study

In total, 216 differentially expressed genes were identified after screening using the DAVID database. Among these, 88 genes were upregulated in the ALV-J resistant chickens after screening for significant changes in expression (threshold: corrected *P* < 0.05 and ratio ≥ 1), compared to the expression levels in the susceptible chickens; the expression levels of 128 genes were downregulated. Among the 88 upregulated genes in the ALV-J resistant chickens, four significantly differentially expressed genes (MCM5, YWHAH, COX6A1, GADD45β; *P* < 0.01) were identified (Table 2). After biological pathway analysis, MCM5, YWHAH, GADD45β were found to be involved in cell cycle signaling pathways (Figure 3); GADD45β was located in the center of the biological signal pathway, downstream of the p53 gene and upstream of the PCNA gene, which is involved in the EndoQ-mediated DNA repair process in *Thermococcales* [26].

**Table 2:** The biological functions of four selected genes of interest in the present study

Gene Symbol	Gene name or description	Reported function
MCM5	Minichromosome maintenance complex component 5	1. Involve in meiotic recombination pathway [20] 2. DNA replication, transcription, cohesion, condensation, recombination [21]
YWHAH	Tyrosine 3-monooxygenase/tryptophan 5-monooxygenase activation protein	1. Regulate development and growth (including cell cycle regulation) and regulate the assembly of microtubules [22] 2. Regulate cell division [23]
COX6A1	Cytochrome c oxidase subunit 6A1	Be required for the stability of holoenzyme [24] It deficiency in Alzheimer's disease [25]
GADD45β	Growth arrest and DNA damage inducible beta	1. Be improtant for anti-tumor immune responses and be necessary for full expression of the Th1 lineage-inducing proteins, T-bet, and Eomes [16] 2. An anti-apoptotic protein in B cells and has the tumor suppressor function [18, 19]

### qPCR analysis of differentially expressed genes in ALV-J resistant chickens

**Figure 4:**
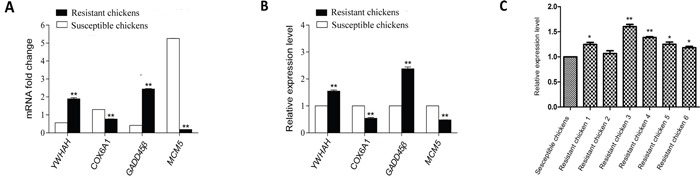
Validation of four differentially expressed genes identified by RNA-Seq with qPCR and identification of GADD45β expression in randomly selected chickens by qPCR **A.** mRNA fold-changes; **B.** relative expression levels; **C.** relative expression of GADD45β in randomly selected chickens. Data shown are the means ± SE, * *P* < 0.05 and ** *P* < 0.01.

To confirm the validity of the four significantly differentially expressed genes of interest that we identified in the ALV-J resistant chickens (compared with the susceptible chickens) by RNA-Seq, quantitative PCR (qPCR) was carried out. The four genes presented consistent trends with the results of the RNA-Seq analysis (Figure 4A, 4B).

### qPCR analysis of GADD45β expression level in randomly selected chickens

qPCR was carried out to identify whether GADD45β was commonly upregulated in ALV-J resistant chickens. We analyzed its mRNA expression levels in another six randomly selected ALV-J-resistant and six ALV-J-susceptible chickens.GADD45β was upregulated in all the ALV-Jresistant chickens compared with the ALV-J susceptible chickens (Figure 4C).

### Effect of overexpression of GADD45β and COX6A1 on ALV-J replication in DF-1 cells

**Figure 5:**
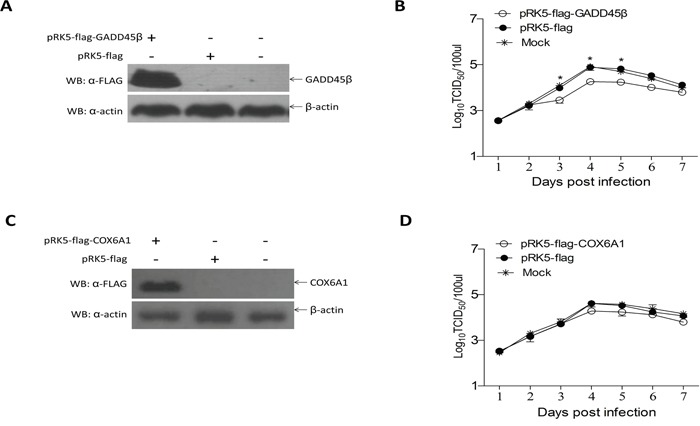
Effects of GADD45β and COX6A1 overexpression on ALV-J replication **A** and **C.** Western blot of the proteins isolated 24 h after transfection of DF-1 cells with the GADD45β or pRK-5 vector, and for the normal cells control group (n = 3), and after transfection of cells with the COX6A1 overexpression plasmid or pRK-5 vector, and for the normal cells control group (n = 3). **B** and **D.** Proliferation curve of ALV-J virus in DF-1 cells transfected with the GADD45β and COX6A1 overexpression plasmids respectively and challenged with ALV-J; * *P* < 0.05.

To identify whether overexpression of GADD45β and COX6A1 proteins in ALV-J-susceptible cells could affect ALV-J replication, we constructed the eukaryotic expression plasmids for pRK5-flag-GADD45β and pRK5-flag-COX6A1 and these were respectively transfected into DF-1 cells. The viral titer during the 7 days after inoculation with ALV-J demonstrated that the expression of GADD45β protein decreased replication of ALV-J, compared with cells transfected with pRK5-flag and a negative control (Figure 5A, 5B). However, the viral titer during the 7 days after inoculation with ALV-J demonstrated that the expression of COX6A1 protein could not effect ALV-J replication (Figure 5C, 5D).

### Effects of SiRNA targeted to GADD45β on ALV-J replication in DF-1 cells

**Figure 6:**
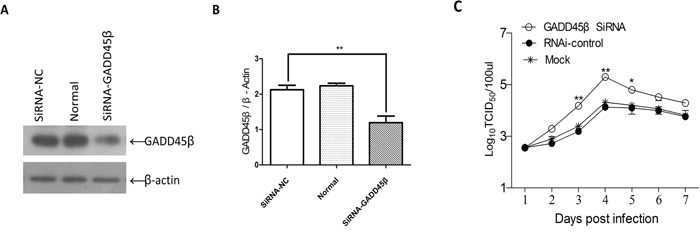
Effects of interference with expression GADD45β on ALV-J replication **A.** Western blot of the proteins isolated 24 h after transfection of DF-1 cells with SiRNA targeted to GADD45β, for the pRK-5 vector and for the normal cells group (n = 3). **B.** Quantities (GADD45β/β-actin ratio) of the isolated proteins shown in (A) determined using Image-Pro Plus 6.0; **P < 0.01 (very significant difference). **C.** Proliferation curve of ALV-J in DF-1 cells transfected with the SiRNA for GADD45β and challenged with ALV-J; *P < 0.05, **P < 0.01.

To evaluate whether low expression of GADD45β would promote ALV-J replication in ALV-J susceptible cells, we transfected DF-1 cells with SiRNA for GADD45β and then tested the effects on the ALV-J replication. The viral titer for 7 days after inoculation with ALV-J showed that the SiRNA for GADD45β efficiently promoted ALV-J replication, especially 3, 4 and 5 days after inoculation. The viral titer was highest 4 days after inoculation (Figure 6).

## DISCUSSION

ALV-J is associated with diverse pathotypes and can result in enormous economic losses; as such, ALV-J has become one of the most important problems facing the global poultry industry [27]. In China, ALV-J infections of broilers were first detected and reported in 1999 [28]. ALV-J has since spread to many countries, such as the United States, Japan, and Israel [29, 30, 31]. Dead or sick birds generally have hemangiomas of various sizes distributed on the surface of their heads, claws, and wings. Some infected birds have gray–white nodules in the liver, spleen, or kidneys, and the liver and spleen can be enlarged to several times their normal size [32].

Developed countries and China have made efforts to eradicate ALV-J from chicken breeding flocks in the poultry industry [33]. Unfortunately, an effective vaccine against ALV-J infection is not currently available [34]; therefore, purifying pedigree generations has become a priority in the primary breeder industry. Breeding chickens that are naturally resistant to ALV-J infections is a new prevention and control measure being used. Through the present study, we have successfully identified a chicken line (line A) that has high congenital resistance to ALV-J infection and cultivated ALV-J infection resistant chickens through a method involving a series of strict screening steps.

RNA-Seq offers the ability to discover new genes and transcripts, and measure transcript expression levels in a single assay [35, 36, 37]. The number of reads produced from an RNA transcript is a function of that transcript's abundance, and the read density can be used to measure transcript and gene expression levels [37, 38, 39]. Expression levels are frequently used to characterize gene functions and can be analyzed using northern blotting, PCR, microarrays, and, more recently, RNA-Seq [40]. Our study used RNA-Seq technology to analyze gene expression levels and identify the genes involved in resistance to ALV-J infections; qPCR was used to verify the accuracy of the GADD45β RNA-Seq results.

A previous study demonstrated that GADD45β was an important signaling modulator for immune surveillance [16]; therefore, we wanted to explore whether the protein was essential in the differing outcomes of ALV-J infection that occurred between the ALV-J-resistant and -susceptible chickens. In DF-1 cells, we overexpressed and interfered with GADD45β expression, then measured the viral titer every day for 7 days. We were surprised by the viral titer measurements, which were lower in the DF-1 cells with overexpression of GADD45β and higher in the cells with disrupted expression of GADD45β (compared to the controls). The research results adequately demonstrate the role of GADD45β in ALV-J replication. We conclude that GADD45β was upregulated in ALV-J resistant chickens and could enhance their defense against ALV-J infection. In addition, we confirmed by qPCR that GADD45β was consistently up-regulated in those randomly selected chickens resistant to ALV-J infection. Thus, GADD45β could be used as a molecular marker of ALV-J infection.

Among the four significantly differentially expressed genes that we selected, the GADD45β and YWHAH (upregulated), and MCM5 (downregulated) genes were involved in cell cycle signal pathways. The COX6A1 (a downregulated gene) encodes a membrane protein not involved in the cell cycle signal pathways; because previous study has demonstrated that it is associated with disease [25], we also researched its relationship with ALV-J replication. We constructed a eukaryotic expression plasmid (pRK5-flag-COX6A1) and overexpressed COX6A1 in DF-1 cells. However, its overexpression did not affect viral replication. Therefore, we concluded that COX6A1 was not a key player in the outcome of the viral infection. Considering that the YWHAH gene had high expression levels in ALV-J resistant chickens and is involved in cell cycle biological pathways, we plan in future research to identify whether it is another anti-disease gene and explore its functions in relation to ALV-J replication.

In conclusion, we have bred ALV-J resistant chickens, and then screened and identified an anti-viral gene, GADD45β. Overexpression of or interference with GADD45β in DF-1 cells significantly affected viral replication. Our findings contribute to understanding of why ALV-J resistant chickens are able to stop ALV-J infections. GADD45β mRNA could also be used as biomarker to detect resistance to ALV-J infection in chickens.

## MATERIALS AND METHODS

### Ethics statement

The experiments were carried out strictly in accordance with the recommendations of the Guide for the Care and Use of Laboratory Animals of the National Institutes of Health. The use of animals in this study was approved by the South China Agricultural University Committee of Animal Experiments (approval ID: 201004152).

### Virus and cell lines

The standard ALV-J NX0101 strain which induced myeloid tumors was used in this study. DF-1 cells, an immortalized chicken embryo fibroblast cell line, were cultured in DMEM media supplemented with 10% fetal bovine serum (FBS; Invitrogen Gibco Co., Carlsbad, CA, USA).

### Resistance of different chicken lines to ALV-J infection

Around 800 one-day-old chickens in eight different lines (A, B, C, D, E, F, G, H; about 100 per line) were used. Chickens were given an intraperitoneal inoculation of the NX0101 isolate (100 μL/chicken, containing 10^3.6^ TCID_50_/0.1 mL). The virus was cultured in DF-1 cells and viral titers were calculated using the 50% tissue culture infective dose (TCID_50_), according to Reed and Muench [41]. Whole blood and anticoagulant blood post-challenge was collected for detection using reverse transcription PCR (RT-PCR), virus isolation, P27 group specific antigen detection by ELISA, and antibody detection, every 2 weeks. The positive infection rate of each chicken line was calculated to identify the most resistant line to ALV-J infection.

### Cultivation of ALV-J infection-resistant chickens

The chicken line that had the lowest positive infection rate for ALV-J was chosen. One cock and one hen from the chicken line were chosen, challenged with ALV-J and then controlled mating was performed. RT-PCR detection, virus isolation, ALV-P27 antigen capture ELISA, and antibody detection were used to detect the positive infection rates from the newly hatched chickens every 2 weeks. The chickens that tested positive for ALV-J were removed and one cock and one hen from the chickens that were not infected were challenged with ALV-J and then used for further controlled mating. The positive infection rates of the offspring (F1 generation) were again determined. One cock and one hen from the chickens in the F1 generation that tested negative (one-day-old) for ALV-J were challenged with ALV-J and used for controlled mating. The newly hatched chickens (F2 generation) that tested positive for ALV-J were removed and one cock and one hen from the F2 generation that tested negative (one-day-old) were again challenged with ALV-J and used for controlled mating to obtain the F3 generation. Challenges with ALV-J were done via intraperitoneal inoculations containing 10^5.5^ TCID_50_/0.1mL. Livers of three chickens from the F3 generation that tested negative by IFA after ALV-J infection, and three that tested positive, were collected for RNA-Seq analysis.

### RNA-Seq

Total RNA was isolated from each of the liver samples collected from the chickens that were respectively resistant and susceptible to ALV-J infection; TRIzol reagent was used according to the manufacturer's instructions (Life Technologies, USA). The RNA samples from within each group (resistant and susceptible) were pooled in equal amounts. The total RNA samples were quantified using electrophoresis (Agilent Bioanalyzer 2100). In preparation for the RNA-Seq, mRNA enrichment, fragmentation, cDNA construction, terminal repair, adding A at the 3′ end, coupling connection, PCR amplification, quality control of the cDNA library, standardization of the cDNA library, and cluster generation was undertaken. Sequencing was performed on an Illumina Hiseq 2500 platform.

The raw reads (fastq format) from the RNA-Seq data were obtained and then cleaned (fastq). The cleaned reads were aligned with the chicken reference genome, LncRNA, and went to cSNV calling. Expression at the gene level was analyzed and the total number of reads, mapped to their unigenes, were calculated and normalized to FPKM (fragments per kilobase of transcript per million mapped reads). Differentially expressed genes were identified with the R package DEGseq, using a Benjamini q-value of 0.05 (cut-off at 5% false discovery rate [FDR]). Differentially expressed genes were selected using the following criteria: fold change ≥ 2 or ≤ 0.5, and *P* < 0.05. The DAVID database was used to perform KEGG and Go enrichment analyses, with the GOseq package, using a corrected *P*-value of < 0.05, and ratio ≥ 1 as the threshold. Differentially expressed genes of interest were selected according to their functions.

### RT-PCR and qPCR (quantitative real-time PCR)

RT-PCR was used to confirm the mRNA expression levels determined using RNA-Seq. Total RNA was extracted from each of the livers dissected from the chickens respectively resistant and susceptible to ALV-J infection using a commercial RNA extraction kit (QIAamp RNA Stool Mini Kit, QIAgen, Hilden, Germany), according to the manufacturer's instructions. For the mRNA expression analysis, cDNA synthesis from the mRNA was performed using a PrimeScript RT Reagent Kit (Perfect Real Time, TaKaRa). The qPCR primers were designed in Oligo 6.0. The GAPDH gene was used as an internal control. Reverse transcription was performed using a ReverTra Ace qPCR RT Kit (Toyobo). qPCR was performed on an Applied Biosystems 7500 Fast Real-Time PCR System (Roche, Switzerland; software version 2.0.5) with a SYBR Green qPCR Mix (ROX) (Roche, Switzerland). The 2^−ΔΔCt^ method was used to analyze the results of the qPCR [42].

### qPCR analysis of GADD45β expression level in randomly selected chickens

qPCR was carried out to identify whether GADD45β upregulation commonly occurred in ALV-J-resistant chickens, we analyzed the mRNA expression level in six randomly selected ALV-J-resistant and six ALV-J susceptible chickens. Blood of each chickens was collected and total RNA was extracted. The 2^−ΔΔCt^ method was used to analyze the results of the qPCR as described above. We calculated the mean Ct in the ALV-J susceptible chickens and the mean value was used to calculate the GADD45β expression level in the ALV-J resistant chickens.

### Vector construction

To construct the pRK5-flag-GADD45β and pRK5-flag-COX6A1, PCR amplification of the GADD45β mRNA and COX6A1 mRNA was conducted and then mRNAs of GADD45β and COX6A1 were cloned into the pRK5-flag vector respectively, using SalI and Bam HI sites for GADD45β and Bam HI and Hind III sites for COX6A1. The primers for the GADD45β mRNA PCR were: 5′-GGATCCATGACTCTGGAAGAGACGCA-3′ and 5′-GTCGACTCACTCAGGTAAGGCAATAGTTGG-3′. The primers for the COX6A1 mRNA PCR were: 5′-GGATCCATGGCGGCGCTCAGAGGG-3′ and 5′-AAGCTTTCACCCCAGGGAAAAGGC-3′. Vectors pRK5-flag-GADD45β vector and pRK5-flag-COX6A1 were sequenced; the predicted nucleotide sequences were successfully identified.

### Effects of overexpression of pRK5-flag-GADD45β and pRK5-flag-COX6A1 on ALV-J replication

DF-1 cells were seeded in 12-well plates. When the cells grew to 80% confluence, they were transfected with 2 μg of pRK5-flag-GADD45β, pRK5-flag-COX6A1 or pRK5-flag vector using Lipofectamine 2000 reagent (Thermo Fisher Scientific). After incubation for 4 h, the Lipofectamine 2000 transfection reagent was removed, and the cells were replenished with DMEM media supplemented with 2% fetal bovine serum. After 24 h, the cells were inoculated with 50% tissue culture infectious doses (TCID_50_) of ALV-J (NX0101). After a further 6 h, the cells were replenished with DMEM medium with 10% fetal bovine serum. The inoculation period was 7 days; seven plates of cells transfected with pRK5-flag-GADD45β and seven plates of cells transfected with pRK5-flag-COX6A1 were prepared. One plate from each group was collected every day and stored at −80°C. Each group's TCID_50_ was then measured.

### Effects of SiRNA targeted to GADD45β in DF-1 cells on ALV-J replication

SiRNA targeted to GADD45β and the negative control SiRNA were synthesized by GenePharma (GenePharma). The SiRNA for GADD45β sequence was 5′-CCAGAUAACGUGGCGUUCUTT-3′ and 5′-AGAAC GCCACGUUAUCUGGTT-3′; the negative control SiRNA sequence was 5′-UUCUCCGAACGUGUCACGUTT- ′ and 5′-ACGUGACACGUUCGGAGAATT- 3′. DF-1 cells were transfected with 2 μg SiRNA for GADD45β or negative control SiRNA. The effects of the SiRNA on GADD45β expression were analyzed through western blot, using anti-GADD45β (AV48346, Sigma-Aldrich). Before transcription, DF-1 cells were seeded in 12-well plates. When the cells grew to 50% confluence, the DMEM medium with 10% fetal bovine serum was removed and DMEM medium without fetal calf serum was added. After 1 h, the DF-1 cells were transfected with 2 μg of SiRNA for GADD45β or negative control SiRNA, using X-tremeGENE SiRNA Transfection Reagent (Roche Life Science). Three replications of each treatment were performed. After incubation for 4 h, the transfection reagent was removed, and the cells were replenished with DMEM medium, supplemented with 2% fetal bovine serum. After 24 h, the cells were inoculated with 50% tissue culture infectious doses (TCID_50_) of ALV-J (NX0101). After a further 6 h, the cells were replenished with DMEM medium, with 10% fetal calf serum. The inoculation period was 7 days; seven plates were set up and one plate was collected every day and then stored at −80°C. Each group's TCID_50_ was then measured.

### Western blot analysis

DF-1 cells were subjected to western blot analysis as previously described by Chen et al. [43]. Briefly, the primary antibodies used were polyclonal rabbit anti-GADD45β (1:500; predicted molecular weight: 18 kDa; Sigma, USA), polyclonal rabbit anti-COX6A1 (1:500; predicted molecular weight: 10 kDa; Sigma, USA) and β-actin (1:1000; predicted molecular weight: 42 kDa; Bioss Inc.), which was used as a protein-loading control. The secondary antibody was goat polyclonal anti rabbit IgG (H+L)-horseradish peroxidase (HRP; Bioss Inc).

### Indirect immunofluorescence assay (IFA)

Indirect immunofluorescence assay (IFA) was performed on the DF-1 cells. The monoclonal antibody JE9, which is specific to the gp85 gene of ALV-J was used as the primary antibody [44]. FITC-goat anti-mouse IgG was used as the secondary antibody.

### Statistical analysis

Data were processed using GraphPad Prism (version 5.0) and are expressed as the mean ± SE. The Student's t-test was used to assess differences among groups, where *P* < 0.05 and *P* < 0.01, were considered to show significant differences between the groups. Western blot bands were quantified with Image-Pro Plus 6.0.
